# Evolutionary effects of fishing gear on foraging behavior and life‐history traits

**DOI:** 10.1002/ece3.4482

**Published:** 2018-10-25

**Authors:** Marion Claireaux, Christian Jørgensen, Katja Enberg

**Affiliations:** ^1^ Institute of Marine Research Bergen Norway; ^2^ Department of Biological Sciences University of Bergen Bergen Norway

**Keywords:** behavior, boldness, fishing‐induced evolution, foraging rate, life‐history traits, mortality, timidity

## Abstract

Fishing gears are designed to exploit the natural behaviors of fish, and the concern that fishing may cause evolution of behavioral traits has been receiving increasing attention. The first intuitive expectation is that fishing causes evolution toward reduced boldness because it selectively removes actively foraging individuals due to their higher encounter rate and vulnerability to typical gear. However, life‐history theory predicts that fishing, through shortened life span, favors accelerated life histories, potentially leading to increased foraging and its frequent correlate, boldness. Additionally, individuals with accelerated life histories mature younger and at a smaller size and therefore spend more of their life at a smaller size where mortality is higher. This life‐history evolution may prohibit increases in risk‐taking behavior and boldness, thus selecting for reduced risk‐taking and boldness. Here, we aim to clarify which of these three selective patterns ends up being dominant. We study how behavior‐selective fishing affects the optimal behavioral and life‐history traits using a state‐dependent dynamic programming model. Different gear types were modeled as being selective for foraging or hiding/resting individuals along a continuous axis, including unselective fishing. Compared with unselective harvesting, gears targeting hiding/resting individuals led toward evolution of increased foraging rates and elevated natural mortality rate, while targeting foraging individuals led to evolution of decreased foraging rates and lower natural mortality rate. Interestingly, changes were predicted for traits difficult to observe in the wild (natural mortality and behavior) whereas the more regularly observed traits (length‐at‐age, age at maturity, and reproductive investment) showed only little sensitivity to the behavioral selectivity.

## 1 INTRODUCTION

Even though most food production is now taking place under controlled conditions in farms, fishing is one exception where we rely on wild populations reproducing in their natural habitat, although we exploit them with industrialized technology and efficiency. The traits of wild fish are therefore still subjected to natural selection and may in addition evolve in new directions as they experience selective pressures from fishing and other human activities (Law & Grey, 1989). Identifying these selective drivers and understanding their impact on the evolution of wild populations are necessary for ensuring long‐term productivity and sustainability of living resources.

Because fishing gears are designed to exploit the natural behaviors of fish, the concern that fishing may cause evolution of behavioral traits has been receiving increasing attention (Arlinghaus et al., 2017; Biro & Stamps, 2008; Cooke, Suski, Ostrand, Wahl, & Philipp, 2007; Diaz Pauli & Sih, 2017; Leclerc, Zedrosser, & Pelletier, 2017; Uusi‐Heikkilä, 2008; Wilson, Clark, Coleman, & Dearstyne, 1994). A key concept in behavioral studies is boldness, defined by placing an individual's level of risk acceptance during behaviors such as foraging, exploration, and defense along a boldness–shyness continuum (Budaev, 1997; Sih, Bell, Johnson, & Ziemba, 2004; Wilson, 1998; Wilson et al., 1994). Risk‐taking is often consistent among contexts and situations, and correlated to other traits. For example, individuals more willing to inspect novel objects will also tend to take risks in other settings and move around more. These correlation structures have been termed “animal personalities” or “behavioral syndromes” (Sih et al., 2004; Wilson et al., 1994). Therefore, boldness is not a single trait, but rather a label ascribed by experimenters and modelers to typically co‐occurring behavioral traits.

Several studies have found that actively foraging individuals may have higher encounter rates with “passive” fishing gears such as gillnets, traps, and baited hooks and will therefore be selectively removed (Biro & Post, 2008; for trout; Philipp et al., 2009; for largemouth bass *Micropterus salmoides*; Biro & Sampson, 2015; for Australian common yabby, *Cherax destructor*; Diaz Pauli, Wiech, Heino, & Utne‐Palm, 2015; for guppies *Poecilia reticulata*). As foraging is a trait typically related to boldness, it is often correlated to other behaviors that are consistent across contexts and situations (Dochtermann, Schwab, & Sih, 2015; Mousseau & Roff, 1987; Sih et al., 2004). The removal of actively foraging individuals, generation after generation, may therefore lead to evolution of multiple traits over time, in what can be summed up as evolution of reduced boldness. This type of selection also takes place when some fish more efficiently escape “active” or moving gears such as trawls (Diaz Pauli et al., 2015).

The expectation, as argued by numerous studies and recently reviewed by Arlinghaus et al. (2017), is that passive gears eliminate bold individuals from the existing trait variation in the population, which causes the evolution of reduced boldness, that is, timidity, over time. Thus, when Arlinghaus et al. (2017) and other studies claim that boldness will decrease due to fishing, the prediction is not that an individual, in the rare case it may encounter a novel object, will approach it more slowly. Instead, the focus is on the behavioral syndrome and that fishing gear, because it exploits certain behaviors related to boldness, may have consequences for correlated traits such as foraging, survival, and in turn population dynamics, trophic interactions, and fisheries yield (Arlinghaus et al., 2017; Biro & Stamps, 2008; Uusi‐Heikkilä, 2008). In the remainder of this article, we use the term “boldness” interchangeably with the level of foraging activity, which in our model is the behavior that leads to ingestion of food while at the same time exposing individuals to predation risk. In the model, fish find more food if they are more active, but they also run into predators more frequently, which are common elements of the “bold” behavioral syndrome. Shyer fish have been found to have lower metabolic rates (Cooke et al., 2007; largemouth bass), lower energetic requirements (Cutts, Metcalfe, & Taylor, 2002; Atlantic salmon *Salmo salar*; Cooke et al., 2007; and Nannini, Wahl, Philipp, & Cooke, 2011; largemouth bass), and more efficient energy conversion (Nannini et al., 2011; largemouth bass). Selection on boldness can further have amplified effects on fitness through reproductive behaviors, for example in largemouth bass where Sutter et al. (2012) documented how males that were more vulnerable to angling also were more aggressive and more active in parental care. This body of studies makes compelling arguments that direct selection on foraging behavior will cause evolution toward reduced boldness with potential consequences including reduced growth, reproduction, population resilience, and fisheries productivity.

However, life‐history theory predicts that fishing, like other sources of external mortality, mostly selects toward early maturation (Law & Grey, 1989) and accelerated life histories (e.g., faster juvenile growth and smaller length at maturation; see Heino & Godø, 2002 for a detailed discussion). Because life‐history traits and behavior are correlated, the general expectation is that accelerated life histories are also associated with a riskier behavior to acquire the resources needed to sustain it (Fraser & Gilliam, 1987; in guppies, *P. reticulata* and Hart's rivulus, *Rivulus hartii*; Biro & Stamps, 2008; Réale et al., 2010). We may therefore expect the elevated mortality from fishing to lead to riskier foraging and bolder individuals over evolutionary time. Even though risky behavior might increase mortality further, it is more beneficial for individuals, under these conditions, to prioritize immediate reproductive gains over long‐term survival or future reproduction (Jørgensen & Holt, 2013; Werner & Anholt, 1993). This argument thus reaches the opposite conclusion but, being complex and involving multiple traits, it is not as verbally persuasive.

The story does not stop there, however, because there is even another layer of feedbacks at which life‐history traits can have effects. Earlier onset of reproduction is well documented as a main effect that is easily detected in fisheries data (reviewed by Heino, Díaz Pauli, & Dieckmann, 2015), and many exploited fish stocks now have smaller body size than before. Because natural mortality declines with size (Gislason, Daan, Rice, & Pope, 2010), these early‐maturing fish spend more of their life at a smaller size where mortality is higher. These fish therefore run into higher risks when foraging, which can prohibit further increases in boldness, simply because the cumulative risk would be too high.

Fishing may thus typically cause three opposing selection patterns for behavioral boldness: direct selection on behavior for reduced boldness; indirect selection through reduced expected life span for increased boldness; and a further route of indirect selection from smaller body size for decreased boldness (Table 1). Which of these three selective forces acting on boldness ends up being numerically dominant likely depends, among other factors, on the type of selectivity of the fishing gear (exactly how accurately does it target behaviors associated with boldness) and the selectivity and level of the other sources of natural and harvesting mortality.

**Table 1:** Schematic illustration of how fishing gear selectively removing bold individuals may affect evolution of boldness when additional layers of life‐history feedback mechanisms are included

Certain types of fishing gear are selectively removing the boldest individuals…		
	▼	
**Expectation** ↓**:** Boldness will evolve to lower values because it is a heritable trait**Applies to:** Verbal arguments in, for example, Biro and Stamps (2008), Arlinghaus et al. (2017). Likely outcome in single‐trait experiments **Conclusion:** Boldness cannot be seen in isolation but requires a lifetime integration, and this perspective is therefore likely not applicable to the wild	**…but fishing is also reducing life span…**	
		▼
	**Expectation** ↑**:** Boldness will evolve to higher values because increased mortality favors early reproduction and accelerated life histories **Applies to:** The model by Andersen et al. (2018), which finds selection gradients on lifetime fitness one trait at the time **Conclusion:** Possible transient or short‐term effect, because behavior may change or evolve faster than body size	**…and it causes evolution of earlier maturation and smaller size**
		**Expectation** ↓**:** Boldness will evolve to lower values because size‐dependent predation is higher for smaller‐bodied fish, prohibiting increases in risk‐taking and boldness **Applies to:** Andersen et al. (2018) (evolutionary transients) and the model and results in this paper (evolutionary endpoints) **Conclusion:** Includes all evolutionary feedbacks, but predictions depend on methodology

Some evolutionary models have already included effects on both life‐history traits and behavior (although rather rudimentarily), and these made the prediction that the risk‐taking during foraging, a characteristic of boldness, would increase slightly due to fishing (Jørgensen & Fiksen, 2010; Jørgensen & Holt, 2013). This expectation was recently analyzed by Andersen, Marty, and Arlinghaus (2018) in a model where fishing selects on boldness, and fitness is quantified as the expected lifetime reproductive output. The authors interpreted the model as predicting reduced boldness, that is, inducing a timidity syndrome (Arlinghaus et al., 2017). However, while selection toward reduced boldness was true for some parameter combinations, selection toward increased boldness over time took place in most of the parameter space explored (see their figures 4b and 6).

In this study, we present a model that in some respects resembles that of Andersen et al. (2018), although the models and analyses have been developed independently and in parallel. Because the models differ in assumptions and evolutionary methodology, the degree of shared predictions makes a stronger case for how behaviors may evolve due to fishing, while the differences in predictions can be traced back to model‐specific assumptions. We aim to clarify theoretical expectations for how fishing activities are selective for behavioral traits, and what consequences are for the evolution of risk‐taking behavior, life‐history traits, and emergent natural mortality.

## 2 MATERIALS AND METHOD

### 2.1 Model description

To assess the impacts of behavior‐selective fishing on behavioral and life‐history traits, we adopted a state‐dependent dynamic programming model (based on Jørgensen & Fiksen, 2010; see also Mangel, 1994; Satterthwaite et al., 2009). The model finds optimal lifelong trajectories for foraging, growth, and reproduction. The new element of this version is that we focus on how fishing gears select on behavior along a continuous axis with two different gear types at each end of the spectrum.

In the one end, individuals are vulnerable to fishing gear when actively looking for food or foraging (e.g., gill nets, lures, baited hooks). We will refer to this fishing situation as targeting the “foraging individuals.” In the other end of the spectrum, individuals are vulnerable when they are not actively foraging. As an example, purse seines might be selecting individuals that are seeking shelter in the safety of the school (Hamilton, 1971; Krause, Bumann, & Todt, 1992) whereas individuals on the outskirts, where more food is available, might have more chance of escaping the gear. We will call this gear type targeting the “resting/hiding individuals.” It is important to note that it is difficult to place precisely the above‐mentioned gears on our continuum because it depends on the gear and on the biology of the targeted species. The gears mentioned here are therefore used as an illustration of a concept.

For more clarity, we deliberately avoid the use of “active” and “passive” gear. “Passive” gears are usually defined as catching the fish as a result of the movement of the fish toward the gear and are also considered as stationary (the opposite is true for active gears, Cochrane & Garcia, 2009). However, we see this view as confusing in the context of our study. For example, trawling is considered as an active gear but can target both foraging and resting fish, depending on where and when it is deployed and, therefore, be located at both ends of the continuum we model.

Most gear types select simultaneously for several, both behavioral and morphological, traits. For simplicity and ease of interpretation of results, our model is selecting purely on foraging behavior, and we exclude size selectivity from the current analysis to avoid confusing the effect of behavioral selectivity with the already complex effects of selection on body size (e.g., Jørgensen, Dunlop, Opdal, & Fiksen, 2009; Zimmermann & Jørgensen, 2017). Excluding size selectivity will also allow us to disentangle the effects of fishing mortality and selectivity pattern. Because the model assumes behavioral vulnerability to fishing gear as a continuum, our analysis includes also the case where vulnerability to fishing is not correlated with behavior. Even if this scenario is not likely to occur in reality, it is the assumption used in most previous modeling studies and the one that fisheries management operates with.

The foraging activity affects growth, as well as the individual's exposure to predators and fishing gear. The key trade‐offs are as follows: (a) between energy acquisition and survival, as increased foraging leads to increased exposure or vulnerability to predation and (b) allocation of acquired resources between growth and reproduction. We describe the model briefly below. For further details please confer Jørgensen and Fiksen (2010), Jørgensen and Holt (2013) and the Supporting Information Appendix S1. All model variables are summarized in Table 1 and parameters in Table 2.

**Table 2:** Summary of the variables used in the model. Prefix S denotes equations found in the Supporting Information Appendix S1

Variable	Description	Unit	Equation
*α*	State‐dependent variable: proportion of resources allocated to reproduction	–	S1; S2
*ϕ*	State‐dependent variable: risk acceptance related to foraging	–	(2); (4); S6
*L*	Body length	cm	S3; S5
*W*	Somatic body mass	g	(1); S3; S1; S8
*E*	Food availability	g^1–b^·year^−1^	(2)
*G*	Gonad mass	g	(1); S1; S8
*R*	Net intake	g·year^−1^	(1); S1; S2
*Q*	Gonado‐somatic index: weight of the gonads in relation to the total body weight (including gonads)	–	S7; S8
*γ*	Coefficient for the relation between fishing mortality and foraging strategy	–	(4)
*h*	Net available resources	g^1−b^·year^−1^	(1); (2)

Net energy intake (*R*, g year^−1^) corresponds to the total energy intake subtracted the energetic costs of routine metabolism (e.g., standard respiration, activity):

(1) $$R=h\cdot W^{b}-b_{0}\cdot {\left(W+G\right)}^{a},$$

where *W* and *G*, respectively, are the individual's somatic and gonadal weight (in grams), *b* and *a* are metabolic exponents, and *b*_0_ is a metabolic constant (see Table 2 for parameter values). Net energy *R* is allocated between reproduction and growth according to the allocation parameter *α*, thus determining age and size of sexual maturation and influencing postmature growth rate (Supporting Information Appendix S1, Equations S1 and S2).

Food intake *h* depends on the individual's foraging strategy *ϕ* (i.e., more or less active foraging behavior) and food availability *E*:

(2) h=ϕ·E.

Note that *E* is a normally distributed random value reflecting autocorrelated stochasticity in food availability, with mean *μ*_*E*_ and standard deviation *σ*_*E*_ (Table 2; see also Holt & Jørgensen, 2014). More intense foraging (higher *h*) increases growth rate but also leads to higher mortality risk (see below). Energy allocation *α* and the foraging strategy *ϕ* are state‐dependent; that is, they and are optimized for every combination of the individual states age, length, and current value of food availability.

Total mortality *Z* (year^−1^) is split into five components (all in unit year^−1^) (for more details, see Jørgensen & Fiksen, 2010):

(3) $$Z=M_{\mathrm{fixed}}+M_{\mathrm{size}}+M_{\mathrm{reproduction}}+M_{\mathrm{foraging}}+F,$$

where *M*_fixed_ is a constant background mortality rate, *M*_size_ a component due to size‐dependent predation irrespective of behavior, *M*_reproduction_ a mortality component that increases with more intense reproductive investment, *M*_foraging_ is the component related to foraging behavior, and *F* is the fishing mortality (see below). All the parameters used in the model are summarized in Table 3.

**Table 3:** Summary of the parameters used in the model

Parameter	Description	Value	Unit	Equation
*a*	Metabolic exponent	0.7	–	(1)
*b*	Metabolic exponent	0.7	–	(1)
*b*_0_	Metabolic coefficient	0.3	–	(1)
*q*_ref_	Gonado‐somatic index at which *M*_reproduction_ = *M*_size_	0.2	–	S7
*k*	Length–weight relationship coefficient	0.95	g·cm^−3^	S3
*c*	Size‐dependent mortality coefficient	1.2	year^−1^	S5
*d*	Size‐dependent mortality exponent	−0.75	–	S5
*p*	Cost of carrying gonads exponent	2	–	S7
$\bar{\phi }$	Reference value for the foraging strategy	1.4	–	(4)
*M*_fixed_	Fixed mortality	0.05	year^−1^	S4
*μ*_*E*_	Mean of the distribution of *E*	6	–	–
*σ*_*E*_	Standard deviation of the distribution of *E*	2.5	–	–

Fishing mortality *F* depends on the foraging strategy *ϕ* and is otherwise nonselective (i.e., independent of other traits such as size, age, or maturity status). The strength of the association between *ϕ* and fishing mortality is a continuous variable, which allows us to investigate different gear types and fish ecologies. Fishing mortality *F* is split into two components: (a) an unavoidable component which the individual will experience regardless of its behavior, and (b) the behavior‐dependent mortality component contingent on foraging strategy, where the risk acceptance for the foraging strategy *ϕ* is scaled with a reference value *θ* to adjust the sensitivity of the model to *γ* (described below):

(4) $$F=\left(1-\gamma \right)\cdot F_{0}+\gamma \cdot \frac{\phi }{\theta }\cdot F_{0}.$$

The relative importance of these two components can be expected to vary depending on the type of fishery, fishing gear, and species that is being harvested. We therefore included the parameter *γ* (–1 ≤ *γ* ≤ 1) to describe the effect of foraging on gear exposure. Note that this equation can produce negative values for *F* depending on the ratio *ϕ*/*θ*. This was checked for continuously in our simulations and was not a problem, partly because realistic values for *γ* lie in the range −0.3 to 0.3, and the majority of our results are reported for this range. The biological interpretation of *γ* is compounded by effects of the fishing gear and of the harvested species’ ecology. Parameter *γ* describes both the intensity by which the fishing gear selects on certain behaviors, and the magnitude of unavoidable fishing mortality not dependent on behavior.

### 2.2 Proportion of mortality attributed to behavior

When *γ* = 0, the fishing is completely unselective on behavior, and the probability of being caught is the same for all individuals regardless of their foraging strategy *ϕ*. For values of *γ* close to zero, the avoidable part of the fishing mortality is low compared to the unavoidable part, and the overall fishing mortality is only weakly dependent on behavior. When the absolute value of *γ* is approaching one, the behavior‐dependent part becomes the most important component of the fishing mortality and the vulnerability of the fish to fishing is depending almost exclusively on the foraging strategy *ϕ* adopted.

### 2.3 Behavior targeted by fishing

**Figure 1:**
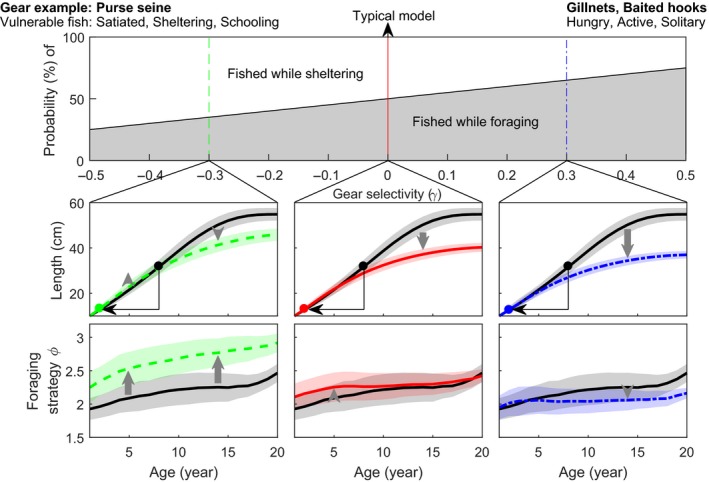
Effect of vulnerability to gear *γ* on optimal life‐history and foraging strategies for fishing mortality of 0.1 year^−1^. In the second row, dots indicate age at first maturation: black ones for populations adapted to natural mortality only, and colored ones after adaptation to fishing mortality (the thin arrow highlights the shift). The solid red line corresponds to the case where fishing is independent of behavior (*γ* = 0). The dashed green and dash‐dot blue lines correspond to the case where fishing targets passive (*γ* = −0.3) and active (*γ* = 0.3) individuals, respectively. Shaded areas correspond to the standard deviation of the trait within the population, and grey arrows show the direction of change due to adaptation to fishing

Parameter *γ* also defines the behavior targeted by the fishery. When *γ* is positive, the vulnerability to fishing increases with the level of foraging activity, and fishing targets individuals with intense foraging strategy *ϕ*. When *γ* is negative, the vulnerability to fishing decreases with the level of foraging activity, and fishing targets individuals with low foraging strategy *ϕ* (illustrated in the top panel of Figure 1).

Because of the association between foraging behavior and vulnerability to different gear types, the realized fishing mortality *F* becomes dependent on the value of *γ*.

### 2.4 Optimization method

The continuous equations above are updated in 24 time steps annually, while strategies *α* and *ϕ* are optimized with annual resolution.

We used optimization by dynamic programming (Clark & Mangel, 2000; Houston & McNamara, 1999) to find the values for foraging strategy and energy allocation that maximized the expected lifetime gonad production. This method thus finds evolutionary endpoints; that is, the evolutionary adaptations one could expect given sufficient time and supposing constraints remained constant (Clark & Mangel, 2000; Houston & McNamara, 1999; Jørgensen & Fiksen, 2010). Finally, we simulated a population following the optimal strategy in an environment with stochasticity in food availability. We ran 10,000 replicates to obtain mean and standard deviation of each trait.

The model was parameterized so that we obtained coherent life‐history traits and trajectories in the absence of fishing, that is, shaped by natural mortality only. Parameter values are summarized in Table 2.

## 3 RESULTS

The model predicts that behavior‐selective fishing induces changes in the optimal foraging strategy of opposite directions depending on which behavior is targeted. In general, when individuals are targeted while foraging, the optimal strategy is to forage less. On the opposite, when individuals are targeted while hiding/resting, the optimal strategy is to spend more time foraging. The qualitative changes observed in other life‐history traits are in line with the general expectations associated with additional mortality: earlier maturation, smaller adult size‐at‐age, and smaller asymptotic size. However, the intensity of these life‐history changes depends on the new optimal foraging strategies induced by behavior‐selective fishing (summarized in Figure 1).

### 3.1 Vulnerability to fishing gear while foraging (*γ)*

**Figure 2:**
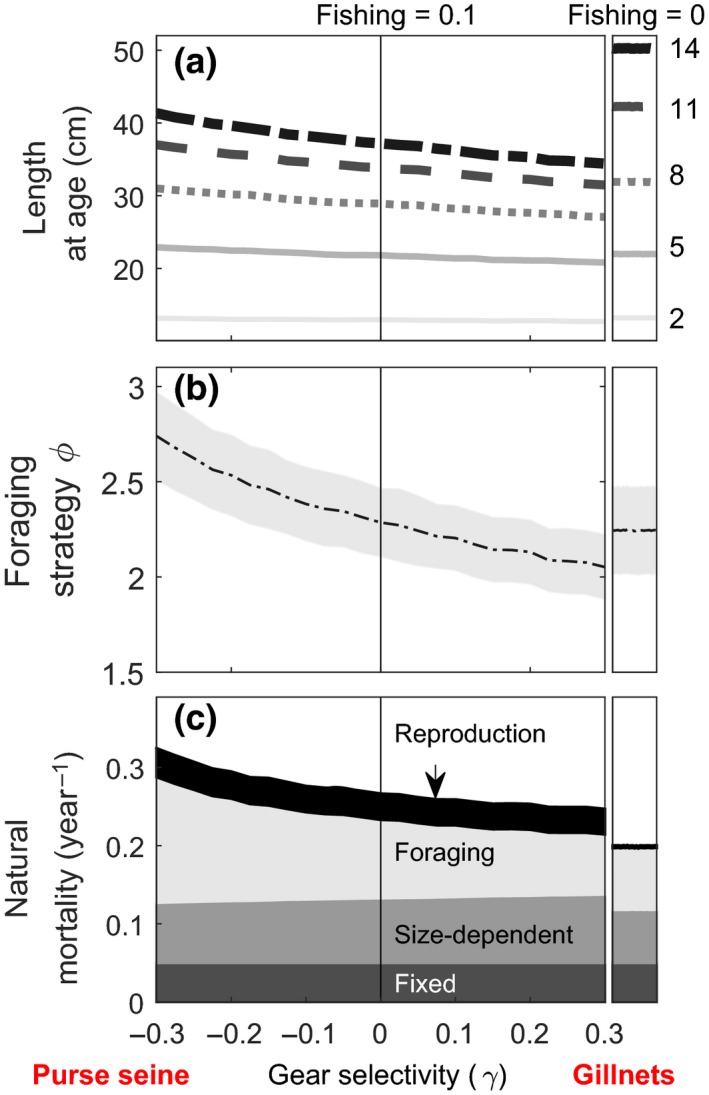
Effect of vulnerability to gear *γ* on predicted optimal values of length‐at‐age (a), the foraging strategy at age 14 (b), and the emergent natural mortality at age 14 (c). The shaded area for the foraging strategy corresponds to the standard deviation of the trait in the population. We use age 14 as the reference for adult life‐history traits because at this age all individuals are mature, even in the absence of fishing. This age is also far enough from the end of the modeled life span to be unaffected by terminal effects when using dynamic programming for optimization (Clark & Mangel, 2000)

Varying the vulnerability to fishing gear while foraging (*γ*) continuously from −0.3 to 0.3 shows clear, directional changes in the optimal life histories, foraging strategy, and emerging natural mortality (Figure 2). Predicted length‐at‐age for adult fish is generally reduced at *F *=* *0.1 year^−1^, but this reduction is more pronounced when fishing targets foraging individuals (Figure 2a). Length at young ages is largely unaffected by the variation in gear selectivity (Figure 2a). The optimal foraging strategy (*ϕ*) is unaffected by unselective fishing (Figure 2b). However, behavior‐selective fishing influences the optimal foraging strategy, and opposite vulnerability (*γ)* have opposite effects: targeting hiding/resting individuals (*γ* < 0) considerably increases the optimal foraging strategy relatively to nonselective fishing (maximum 20% increase), while targeting foraging individuals (*γ* > 0) reduces it (maximum 10% decrease) (Figure 2b). As expected, natural mortality increases, even with unselective fishing (Figure 2c). The model predicts that targeting hiding/resting individuals (*γ* < 0) increases consequently the total natural mortality even further, relative to nonselective fishing (maximum 22% increase). This is particularly due to the component of mortality related to foraging. Targeting foraging individuals (*γ* > 0) results in somewhat lower total mortality rate than nonselective fishing (maximum 7% decrease) (Figure 2c), but still in an increase compared to no fishing.

### 3.2 Interactions between behavioral gear selectivity and the level of fishing mortality

**Figure 3:**
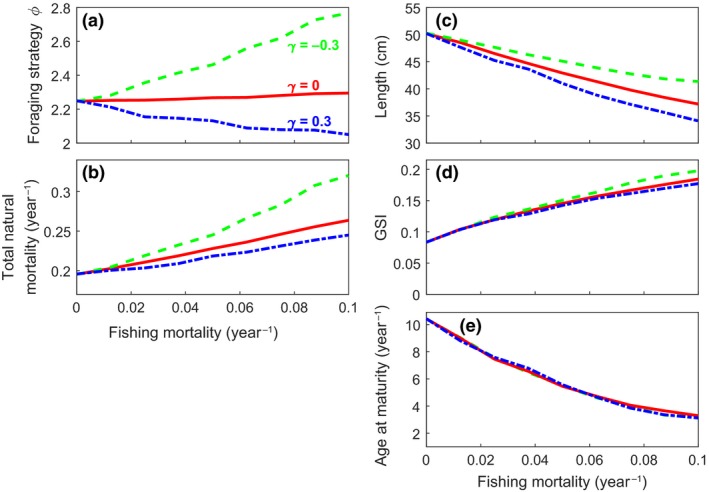
Effects of fishing mortality on average individual‐level traits for different types of vulnerability to gear *γ*. The traits shown are the mean value of the foraging strategy at age 14 (a), total natural mortality at age 14 (note that total mortality includes also fishing mortality which is not shown here) (b), length‐at‐age 14 (c), reproductive investment (GSI) at age 14 (d), and mean age at maturity within a population, depending on *γ*. The red solid lines correspond to the case where fishing is not related to behavior (*γ* = 0). The green dashed (*γ* = −0.3) and blue dot‐dash (*γ* = 0.3) lines correspond to the case where fishing targets sheltering (purse seines) and foraging individuals (gill nets), respectively

The sensitivity of the various life‐history and behavioral traits to intensified fishing mortality depends on the vulnerability to the fishing gear *γ* (Figure 3). We use three distinct selectivity scenarios to exemplify this: (a) targeting hiding/resting individuals (*γ* = −0.3); (b) targeting foraging individuals (*γ* = 0.3); and (c) nonselective fishing (*γ* = 0). Traits can be grouped into two groups based on how sensitive they are to gear selectivity *γ*, and interestingly, this correlates with how easily observable the traits are. The first group consists of traits that are difficult to observe or measure, but where the model predicts a considerable impact of behavioral gear selectivity with intensified fishing (Figure 3a,b). The traits in this group the foraging strategy, which may increase or decrease depending on the vulnerability to fishing gear *γ*, and natural mortality, which increases with intensified fishing for all types of vulnerability *γ* but more when hiding/resting individuals are targeted (*γ* = −0.3; Figure 3b). The second group of traits are more easily observable and measurable, and in many cases already part of standard monitoring of fish stocks and a focus of many models, but are at the same time less sensitive to different gear vulnerability (Figure 3c–e). The traits in this second group are length‐at‐age (Figure 3c), gonado‐somatic index reflecting the reproductive investment (GSI; Figure 3d), and age at maturity (Figure 3e).

Vulnerability to fishing gear while foraging *γ* also highlights changes in energy allocation. When fishing targets foraging individuals (*γ* = 0.3; Figure 3, blue dash‐dot lines), the individual fitness is maximized by reduced activity and adoption of safer foraging strategies (i.e., foraging less, Figure 3a, blue dash‐dot line). The individual survival thus increases through reduced overall natural mortality (Figure 3b), but at the cost of decreased energy acquisition. This leads to less energy being available for growth and reproduction. On the opposite, when fishing targets hiding/resting individuals (*γ* = −0.3; Figure 3 green dotted lines), the survival benefit from safe foraging strategies is reduced. No matter which foraging strategy an individual adopts, it will suffer extra mortality, either from fishing or from predation, and adopting a riskier foraging strategy (i.e., increased foraging rate) appears to be the most optimal solution (Figure 3a). Thus, natural mortality increases, but the positive effect of increased energy acquisition compensates for reduced survival probability (Figure 3b). Investment into reproduction is only little affected by vulnerability to the fishing gear (Figure 3d), but appears to have priority over somatic growth because differences in energy acquisition result in differences in adult length, but not in GSI or age at maturation (Figure 3c–e).

## 4 DISCUSSION

In this study, we analyzed a model which predicted that behavior‐selective harvesting has the potential to alter life‐history traits and emergent natural mortality in addition to the behavioral trait that is directly targeted by the fishing gear. It thus integrates the one direct and two indirect selection routes in Table 1. Our findings support earlier works on evolutionary effects of fishing by predicting earlier age at maturation, increased reproductive investment, smaller asymptotic size, and higher natural mortality (e.g., Dunlop, Enberg, Jørgensen, & Heino, 2009; Enberg, Jørgensen, Dunlop, Heino, & Dieckmann, 2009; Enberg et al., 2012; Jørgensen & Fiksen, 2010; Law & Grey, 1989). Importantly, the model also goes beyond that, in showing that being specific about how gear types interact with a fish species’ ecology has implications for the evolution of behavior (see also Andersen et al., 2018), even when indirect selection via life‐history traits is included.

### 4.1 Fishing can be a driver of reduced boldness

The predictions from our model align with those of Andersen et al. (2018) by showing that behavior may evolve in response to fishing and that different gear types can be selective in different ways. When interpreting these models, it is important to keep in mind that the type of fishing gear and the species’ natural behavior together determine the degree of selection on a trait. For example, many fishing techniques use baits or mimic food, which attracts fish while they are foraging. This will selectively harvest fish that more actively search for food or more indiscriminately eat what they find. Baits and lures may thus be effective in capturing active predators and generalists, of which Atlantic cod can serve as an example. It is thus the combination of species biology and gear type that defines an efficient fishery for cod and, in our model, this leads to decreased boldness (Figure 2, positive *γ*). Andersen et al. (2018) find the same, but only when there is size‐selective harvest of big fish only (their figure 6) and when direct selection on behavior is at the high end of their tested range (their figure 4b). It remains to be argued how typical this combination is, but the clearest example may be rod‐and‐reel angling where lures mimic food and trophy fish are targeted. That our model predicts reduced foraging rates (a characteristic of shyness) for a broader parameter range (as long as fishing gear selectively removes foraging fish) suggests that the phenomenon may occur more widely than suggested by Andersen et al. (2018). The prediction follows from our methodology, where the evolutionary effect of fishing includes reduced body size, at which predation risk is higher to the degree that it prohibits increased foraging rates. The finding is in accordance with the suggestion by Arlinghaus et al. (2017) that passive gears, often selectively removing active individuals, have the potential to induce a “timidity syndrome” in exploited populations.

A further effect that may lead to the evolution of reduced boldness is size‐selective fishing, where fish that mature and stop growing before they reach harvestable size may have higher fitness (see examples in Jørgensen et al., 2009). Reduced foraging may therefore evolve if it can contribute to fish staying small. In our model, foraging activity can vary with age and size, so foraging can slow down as fish reach harvestable size, were we to run such a scenario. This is in contrast to Andersen et al. (2018), where boldness is a lifelong trait and where potential benefits at small size and potential costs at large size are weighed together when selection favors either increased or reduced boldness. With harvesting of large fish, the cost of high foraging when large can outweigh its benefits while being small so that boldness would evolve to become lower, as in their figure 6.

Unfortunately, it is difficult to confront our results to observations from wild populations since behaviors such as boldness and mortality are very difficult to observe and estimate, especially in marine fish stocks. Additionally, selection on the behavior usually comes together with selection on size or other traits and disentangling the effects of both selections from population data would be impossible. Also, in large scale fisheries, gears might not discriminate enough between the different behaviors to introduce a significant effect of behavioral selectivity. To answer this question, we would need more knowledge about quantifying the behavioral selectivity of large scale fisheries gears.

### 4.2 In pelagic, schooling fish, increased boldness is predicted

Where we draw attention to a new concern is in relation to fisheries on schooling fish, as is typical for many pelagic species. A typical gear used is the purse seine, which our model predicted would lead to evolution of higher foraging rates. This is because individual fish seek safety in the school (Hamilton, 1971), and we assume that is where they are more vulnerable to purse seines. To avoid the purse seine, fish could spend more time outside the schools, where foraging is more efficient (Eggers, 1976). Exploitation could thus favor individuals with a higher activity and growth rate, which in turn also leads to elevated natural mortality due to predation. However, even though they are expected to capture shyer fish, it is not yet clear how strong their behavioral selectivity is (Diaz Pauli & Sih, 2017). Our expectations are therefore qualitative, and we cannot infer about how strong the changes in response to this fishing gear will be.

The predicted evolutionary effect of purse seining on behavior could not explain why the Norwegian spring‐spawning herring population (*Clupea harengus*), although extensively monitored and with 80‐year‐long time series of maturation age, shows few signs of earlier maturation (Engelhard & Heino, 2004) in contrast to almost all other stock with a similar exploitation history (Heino et al., 2015). We need to adapt our model precisely to herring before trying to make further conclusions. It is also possible that the behavioral selectivity of the purse seine is, in reality, very low (close to *γ* = 0) and the changes in response to this fishing gear are not detectable in the wild.

As it is, the life‐history modeled is closer to a long‐lived, cod‐like species. Qualitatively, we believe that the general expectations drawn from our model could apply to a wide range of other life histories. However, it is more difficult to predict how our results would change if we decided to model species with a specific ecology inducing additional costs (e.g., extensive migrations, large investments into reproductive behavior). A parameterization of the model specifically for these species would therefore be required before drawing further conclusions on this question. Adaptation toward more risk‐taking phenotypes may also be induced by other active gears such as trawls (Diaz Pauli et al., 2015; guppies; Leclerc et al., 2017).

### 4.3 Similarities and differences with the Andersen et al. (2018) model

Our model is largely similar to that by Andersen et al. (2018) except for four important differences: (a) we focus on finding the optimal values for a set of jointly evolving traits, whereas Andersen et al. (2018) focus on selection responses and selection trajectories; (b) in our model, the behavioral and life‐history traits are varying with age, whereas in the model of Andersen et al. (2018), the trait values are independent of age or size; (c) treat the different types of fishing gears as a continuum rather than specific cases, allowing us to fill a broader canvas; and (d) we ignore the size dependence of fishing gear, in order to favor interpretation and analysis even though it makes the model less applicable to real‐world fisheries. Below we discuss how the approaches differ, and how the contrasting findings can be interpreted.

Our state‐dependent model allows foraging behavior and reproductive investment to be optimized for each age, whereas those traits are assumed fixed to one lifelong trait in Andersen et al. (2018), resulting in a compromise between the optimal trait combinations in different phases of life. In the absence of fishing, we find a slight increase in foraging activity with age. This is because individuals at larger sizes have lower length‐dependent mortality and can afford to forage more, although it makes them more exposed to predators. The shortening of life span caused by fishing leads to decreased age at maturation and increased investment into reproduction. The consequences of these changes are two‐fold: reproduction starts earlier and, consequently, adult fish are smaller, leading to increased size‐dependent predation mortality. When fishing is not selective for behavior (*γ* = 0), the increase in predation mortality due to smaller size prohibits further risk‐taking through elevated foraging, and our model predicts no change in boldness with increasing fishing pressure.

Overall, the age‐dependent changes in foraging activity are rather small in our model, except when resting/hiding individuals are vulnerable to fishing. However, our preliminary runs with size‐selective fishing show that the foraging behavior can vary substantially with age/length when size selectivity is included. This is because adaptations of foraging behavior allow the fish to stay under the target size. For example, with relatively high minimum size, the optimal strategy seems to be foraging less when young in order to stay at smaller size and avoid the fishing mortality, regardless of the behavioral type the fishing is targeting. However, when the size at which fishing starts is reached, the foraging activity will change depending on which behavior is targeted. These preliminary results highlight the importance of including state dependence, especially when introducing a fishery selecting on length, weight, or age.

Different fishing gear can target a range of behavioral as well as physiological traits: Hungry individuals (potentially with high metabolic rate) are more vulnerable to baited hooks (Stoner, 2003), angling is selecting individuals with elevated activity levels and aggressive behavior (Cooke et al., 2007; Suski & Philipp, 2004), and trawls are more efficient in catching fish with a low swimming capacity (Huse, Løkkeborg, & Soldal, 2000), low metabolic rate, and low maximum aerobic swim speed (Killen, Nati, & Suski, 2015). The selection pressures caused by different fishing gears are thus likely to favor different behavioral, physiological, and life‐history strategies, even within the same species. We simplified this into a single continuum describing the strength of correlation between energy acquisition rate (foraging) and vulnerability to fishing mortality. An important difference between Andersen et al. (2018) and our study is that we included the possibility that fishing could target satiated or hiding/sheltering fish, as might be the case with schooling, pelagic fish. It is for these types of fisheries we predict the strongest increases in boldness and, consequently, the most dramatic increases in natural mortality rate.

We excluded size selectivity from our model (fish have the same probability of being caught regardless of size) to avoid confusing the effects of size‐selective harvesting with the ones of behavior‐selective harvesting. The different components of selectivity obviously interact and lead to different trait combinations being optimal under different selectivity combinations. Andersen et al. (2018) analyze this to some degree, but a full treatment of the evolutionary effects of size selectivity is complicated (for cases without behavior, see, e.g., Jørgensen et al., 2009; Zimmermann & Jørgensen, 2017). Comparing the joint effects of size‐ and behavior‐selective fishing is a natural extension of this model but beyond the scope of the current study. We expect the optimal foraging behavior to depend on the type and shape of size selectivity and an additional study dedicated to this specific point is needed.

## 5 MANAGEMENT IMPLICATIONS

Modern fisheries management relies on up‐to‐date estimates of population parameters as an input for realistic stock assessments, and ignoring the evolutionary consequences of fishing might lead to suboptimal management (Biro & Post, 2008; Enberg, Jørgensen, & Mangel, 2010; Heino et al., 2013; Laugen et al., 2014; Uusi‐Heikkilä, Wolter, Klefoth, & Arlinghaus, 2008). Length‐at‐age, reproductive investment, and age at maturity are prone to evolve due to fishery selection, but we showed that the predicted changes are largely independent of behavioral selectivity. At the same time, these are also the most common traits scientists have used to detect fishing‐induced evolution (Heino & Godø, 2002; Law, 2000; Sharp & Hendry, 2009). However, traits crucial for understanding stock dynamics such as behavior and natural mortality are difficult to estimate, and often not estimated at all but out of convenience assumed to be constant, even though notable variations in the latter has been observed in several stocks (Cadigan, 2016; Swain, 2011; Swain & Benoit, 2015; Swain, Jonsen, Simon, & Davies, 2013; Thorley & Andrusak, 2017). Our and previous (Jørgensen & Fiksen, 2010) results suggest that regardless of the behavioral selectivity, natural mortality will increase due to fishing‐induced adaptations, but even more so when fishing targets hiding/resting individuals. Ignoring such increase would lead to underestimation of stock size, even though the fishing mortality maximizing long‐term yield (*F*_MSY_) might not drastically change. Given that in most stock assessment models, the reported catch is the most important entity defining the stock level, while survey time series are used as relative indices, a discrepancy between the observed stock size in the field and the perceived stock estimated by the assessment model might arise. Such discrepancy, where fishermen observe larger amounts of fish than stock assessments estimate, can erode trust, complicate stakeholder dialogue, and in the long run be detrimental for successful management.

Incorporating the effects of behavioral selectivity through different gear types adds to the tool box available for sustainably managing fish stocks in an evolutionarily enlightened manner and potentially mitigating detrimental changes for future fisheries yields as well as population viability. Regardless of the differences in methodology and some differing results when comparing with Andersen et al. (2018), our main findings coincide and make a strong case that behavioral‐selective fishing can induce changes in exploited populations.

## CONFLICT OF INTEREST

The authors declare no conflict of interest.

## AUTHOR CONTRIBUTIONS

MC, CJ, and KE contributed to building the model, interpreting the results, as well as writing and reviewing the article before submission. All authors have approved the final, submitted version.

## DATA ACCESSIBILITY STATEMENT

Script: https://doi.org/10.5281/zenodo.1307394.
