# Supplementary material for: Evolutionary effects of fishing gear on foraging behavior and life‐history traits
Source: Ecol Evol. 2018 Oct 25;8(22):10711–21. doi: 10.1002/ece3.4482 (PMC6262916; doi:10.1002/ece3.4482)
Supplement: Supplementary file 1 [file ECE3-8-10711-s001.docx]

**Evolutionary effects of fishing gear on foraging behaviour and life-history traits**

by Marion Claireaux, Christian Jørgensen and Katja Enberg

# Appendix S1: State-dependent dynamic programming model

***Energy allocation to somatic growth and reproduction***

The energy allocation parameter *α* divides the available resources *R* (net intake in g·year^−1^, Equation 1) between somatic growth and reproductive investment and is state-dependent:

$\frac{dW}{dt}=\left( 1-\alpha\right)\cdot R$ , (S1)

$\frac{dG}{dt}= \alpha\cdot R$ . (S2)

Length (*L* in cm) is isometrically related to weight:

$W=k\cdot L^{3}$ , (S3)

***Mortality***

Total mortality *Z* (y^–1^) is split into five components (all in unit y^–1^) :

$Z= M_{\mathrm{fixed}}+M_{\mathrm{size}}+M_{\mathrm{reproduction}}+M_{\mathrm{foraging}}+F$, (S4)

*M*_fixed_ represents a fixed level of size-, state- and behaviour-independent mortality (e.g., diseases and natural disasters).

*M*_predatio_*_n_* is a size-dependent mortality component, and declines with body size:

$M_{\mathrm{predation}}=c\cdot L^{-d}$. (S5)

Size-dependency assumes that a small fish has more predators than a big one (Werner et al., 1988). The values of c and d can be found in Table 2.

*M*_foraging_ describes the mortality linked to the growth-survival trade-off and is scaled with the *M*_predation_ to follow the size-dependency (Equation 6*). Consequently, *M*_foraging_ depends on the growth strategy *ϕ* adopted. We assume that a risk-prone, active growth strategy is associated with high mortality costs due to increased risk of encountering a predator.

$M_{foraging}=M_{predation}\cdot\phi$. (S6)

The survival cost of reproduction *M*_reproductio_*_n_* is positively correlated with the reproductive investment represented by the gonado-somatic index *Q*. Carrying large gonads is thought to decrease an individual’s swimming ability, lowering the predator escape capabilities (Ghalambor et al., 2004) and thus making the fish more vulnerable to predators. Moreover, *Q* does not only represent the energy invested in gonad production, but also other energetic costs of reproduction. The mortality component associated with reproduction thus also reflects the risks associated with mating behaviour (e.g., while searching for mate or spawning). Reproduction mortality increases with the reproductive investment and is also size-dependent:

$M_{\mathrm{reproduction}}=M_{\mathrm{predation}}\cdot\left( \frac{Q}{q_{\mathrm{ref}}} \right)^{p}$, (S7)

and

$Q= \frac{G}{W+G}$. (S8)

***Optimization***

We used optimization by dynamic programming (Clark and Mangel, 2000; Houston and McNamara, 1999) to find the values for foraging strategy and energy allocation that maximized the expected lifetime gonad production. The growth and allocation strategies are calculated for every length (maximum length is fixed at 200 cm) at every age (maximum age is fixed at 35 years) and for every food environment values. A shortcoming of this method is that we have to work with discrete values of age, length and food environment and cannot have a fully continuous approach. To get around this problem, between t and t+1, mortality rate, size, and gonads are calculated in 24 finer temporal steps and then summed (Jørgensen and Fiksen, 2010; Jørgensen and Holt, 2013).

We define *f*(*a*,*l*) as the fitness of an individual of length *l* at age *a* such as when the individual reaches the maximum age *a*_max_, it dies and so, its future fitness is 0. With dynamic recursive iterations, we can find the maximum fitness and the growth and allocation strategies associated for each age-length-food environment combination (Jørgensen and Fiksen, 2010):

$f\left( a,l \right)={max}_{\alpha,\varphi}\left\{ P\left( E \right)\cdot[s(E)\cdot[g'(E)+f(a+1,l^{'}(E))] \right\}$, (S9)

where *l*’ is the individual length at age *a*+1 and *g*’ the reproduction between age *a* and *a*+1. Parameters *l*’, *g*’ and *s* depend on the environment E. Consequently, the fitness components are summed over the probability of experiencing a given environment E, assuming that the individual adopts an optimal strategy for each value of *γ* (Holt and Jørgensen, 2014). This method thus finds evolutionary endpoints, i.e. the evolutionary adaptations one could expect given sufficient time and supposing constraints remained constant (Jørgensen and Fiksen, 2010, Clark and Mangel, 2000; Houston and MacNamara, 1999). Finally, we simulated a population following the optimal strategy, which is the basis for all results shown.
